# Mapping the multifaceted resilience construct: a facet-based approach

**DOI:** 10.3389/fpsyg.2025.1674912

**Published:** 2025-11-26

**Authors:** Daphna Shwartz-Asher, Aharon Tziner

**Affiliations:** Tel-Hai Academic College, Qiryat Shemona, Israel

**Keywords:** resilience, workplace, multilevel framework, facet analytical approach, conceptualization

## Abstract

This perspective article addresses the profound “definitional crisis” within resilience research, revealing inconsistent findings regarding adversity's role in workplace outcomes. Traditional individual-centric models are critiqued for neglecting systemic factors like racism, colonialism, patriarchy, and capitalism. These models profoundly shape experiences and resource access for marginalized groups. Recent scholarship advocates for multilevel frameworks. Shifting focus from individual traits to community resources, social determinants, and public policy. Furthermore, resilience is conceptualized diversely across disciplines, ranging from dynamic processes to purely economic metrics. Acknowledging these conceptual and methodological limitations, this paper proposes adopting facet analytical approach, as a tentative solution. This framework aims to formalize and clarify the multifaceted resilience construct by breaking it down into four basic facets: (1) Modalities of coping, (2) Time span of resilient behavior, (3) Level of growth, and (4) Domain of outcome. This approach seeks to enhance construct validity and bring much-needed clarity to a theoretically ambiguous field, moving toward a more unified understanding. However, we feel that even this conceptualization approach will not offer a sufficiently adequate solution to the conceptualization of resilience. We suggest a likely solution to this issue.

In their meta-analysis of resilience in the workplace, Good et al. (2025) reported three main findings. First, positive relationships were found among the three components of resilience, capacity, enactment, and demonstration. Next, they found main effect relationships between the three resilience components and the four outcome domains of performance, job attitudes, psychological wellbeing, and physical health. These results support a positive relationship between various resilience constructs and valued workplace outcomes. Finally, referring to several contextual variables (i.e., moderating variables), such as the presence of adversity and occupational risk and the source of outcome measure, the results were less consistent than those concerning main effect variables.

Good et al. (2025) model is an example of a novel theory with inconsistent empirical support. Previously, Britt et al. (2016) observed that past research purporting to study employee resilience suffers from a lack of conceptual clarity about both the resilience construct and the methodological designs that examine resilience without ensuring the occurrence of significant adversity.

This perspective article represents a critical and evolving moment in the study of resilience, exposing a profound lack of a unified theoretical framework. While our literature review engages with the concept of resilience—understood broadly as a positive outcome or adaptation in the face of adversity—the studies delineate, operationalize, and contextualize resilience in such diverse ways that it is clear the field is grappling with a fundamental definitional crisis. Consequently, this paper critiques traditional, individualistic perspectives and pushes for a more complex, multilevel, and structurally nuanced understanding of resilience, ultimately demonstrating that “resilience” is far from a settled theoretical construct.

A dominant theme running through previous studies is the direct challenge to the “incomplete and, at times, damaging story” that mainstream psychology has conveyed about resilience (McLean et al., 2024). McLean et al. argue that traditional models, which often frame resilience as an innate individual trait or as a simple ability to “bounce back,” fail to account for the systemic forces that shape human experience. McLean et al. point out the omission from these conceptualizations of societal systems and structures, such as racism, colonialism, patriarchy, and capitalism, and offer critiques of the current literature using the concept of master narratives to articulate the incomplete and, at times, damaging story that the discipline of psychology has told about resilience.

McLean et al. (2024) critical stance mirrors (Bentley-Edwards and Adams 2024, p. 1036) critique, which discusses how narratives such as #BlackGirlMagic—a social media hashtag and cultural movement that celebrates the beauty, resilience, and achievements of Black women and girls—can paradoxically “glorify struggle, undermine support, and victim-blame.” The implication is that a focus solely on individual (or group) strength without addressing the root causes of adversity can be harmful, forcing individuals to adapt to oppressive conditions rather than challenging those conditions directly. For resilience to be a truly beneficial concept, the term requires clarification on how it is used and understood, particularly concerning marginalized groups. Attempts to imply resilience as an unclear concept, as (Bentley-Edwards and Adams 2024) claimed, can harm the research subjects and perpetuate their vulnerability.

The limitations of individual-centric models are further underscored by the discussion of stigma and access to resources. Notably, King et al. (2024) framework advances interpretations of past work on resilience and posttraumatic growth, their respective conceptualizations and operationalizations, future model development, and interventions. The researchers developed a “stigma-conscious framework” that directly addresses how stigmatization “depleted and blocked” accesses to critical resources like “time, money, energy, and support” (p. 1). The framework signals that existing models of resilience do not adequately account for these systemic barriers, thus hindering their “realism and generalizability” for stigmatized groups. This perspective reframes resilience not as an internal wellspring but as a *process* mediated by external resource availability. That is to say that those affected by lack of external resources (i.e., those labeled as stigmatized) may be harmed by the more traditional ways in which resilience is portrayed in the literature.

Similarly, (Wakeel 2024, p. 1025), referring to resilience among women, critiques the literature for conceptualizing resilience as merely “individual attributes that enable women to ‘bounce back,”' arguing that such an approach fails to incorporate “broader systemic and environmental factors” that contribute to chronic stress, especially among vulnerable communities. Wakeel (2024) insight is particularly relevant given the staggering statistics she cites of women of color who are *at least twice as likely* as non-Hispanic White women to die during the perinatal period or deliver low birthweight or preterm infants. This stark reality necessitates a framework that acknowledges the structural nature of adversity, beyond individual coping mechanisms. In other words, Wakeel (2024) reinforces the fact that there are objective-nonindividual circumstances to be considered that require unique knowledge and expertise when attempting to apply solutions that involve resilience to a distinct social sector.

In response to these critical remarks, several recent studies propose multilevel and multidimensional frameworks. For instance, Del Toro et al. (2024) view resilience as a protective mechanism rooted in familial identity and practices. Walker et al. (2024); Lee et al. (2024); Last et al. (2024) further emphasize the need to expand the scope of resilience beyond the individual to community and societal levels. Walker et al. (2024, p. 1092) specifically examine “community-level resources” that facilitate resilience for Black boys and young men facing “race-based structural inequities and concentrated disadvantage.” Their findings highlight the importance of mentoring relationships and safe spaces, moving the conversation from individual adaptation to community-level intervention. This is echoed by Lee et al. (2024), who propose a “culturally and structurally informed model of resilience that integrates social-ecological and minority stress models.” In that context, Lee et al. (2024) review stressors across multiple levels, from the microsystem (e.g., family rejection) to the macrosystem (e.g., harmful legal policies), arguing that resilience depends on modifiable factors such as “community cohesion” that operate at these different scales. These findings support our claim that resilience as a concept requires a definition that is not simplistic but rather takes into account different external circumstances at both the individual and social sector levels.

The most compelling example of this multilevel approach is the “Social Determinants of Resilience” framework proposed by Last et al. (2024). This conceptual article posits that resilience is fundamentally tied to access to “material and psychological resources,” while indicating that redistributive policies are a primary mechanism for promoting resilience in children and families. The authors examine four U.S. policies—Medicaid expansion, the Earned Income Tax Credit, childcare subsidies, and Temporary Assistance for Needy Families—as concrete examples of how societal resources can mitigate the harmful impacts of adversity. By linking resilience to policy and resource access, this framework drastically shifts the definition of resilience from a psychological trait to a social and economic outcome, further muddying its theoretical waters.

Beyond the social and structural, other studies offer completely different conceptualizations of resilience. Bezek et al. (2024, p. 1123), for example, delve into the neurobiological underpinnings of resilience in youth, defining it not as a single outcome but as a set of “positive outcomes across multiple domains (e.g., social, academic).” Their study, based on a sample of 708 twins, identifies specific neural markers and network organization features associated with resilience. This approach defines resilience through a biological lens whereby the asset is a function of brain network organization, such as “whole-brain functional integration” and “robustness to disruption” (p. 1125). This slant adds a layer of biological complexity to discussions on the nature of resilience that stands in sharp contrast to the socioeconomic and cultural definitions presented elsewhere.

Furthermore, (Bergeman and Nelson 2024, p. 1064) present a “Dynamic Adaptational Process Theory of Resilience (ADAPTOR).” Here, resilience is conceptualized as a continuous process involving a “synchronistic interplay of reserve capacity, adaptation, and consequences.” In this model, individuals “build” resilience by drawing on their capacities to adapt to contextual factors, the process having long-term consequences for health trajectories, such as depression and anxiety. Tested by Ahrens et al. (2024) on a longitudinal sample of 444 participants during the COVID-19 pandemic, this framework presents resilience as a fluid, dynamic process rather than a static state or outcome. The findings of these papers point toward a compensatory relationship between reserve capacity and adaptation, further defining resilience as a complex, interactive process.

So, what is resilience? Is it an individual variable? A social characteristic? Or perhaps even a neurobiological issue? To what extent is it dynamic? All the studies presented so far raise many questions regarding the concept. Notably, previous studies differ even in the measurement of resilience. Sheetal et al. (2024), for example, employs a purely economic definition, measuring resilience within the context of the COVID-19 pandemic as the rate of recovery of “socioeconomic activity” from COVID-19 pandemic, using metrics such as public transportation occupancy and cinema attendance. This manner of discussing resilience is a stark departure from the psychological or neurological definitions so far described. Sheetal et al. (2024) study also identifies unique predictors like “obedience to authority” and the “Protestant work ethic” that are not commonly found in psychological resilience research, suggesting that the very predictors of resilience are dependent on its definition.

Finally, we would concur with the conclusion drawn in Infurna et al. (2024)'s meta-commentary that explicitly states that “conceptual and methodological limitations” in the field [of resilience] “call into question the credibility of existing research” and underscore the need for “multidisciplinary perspectives.” This is an acknowledgment that the field is at a crossroads, steering a “new generation” of research that can clarify the multifaceted nature of adversity and resilience, specifically in marginalized communities.

In conclusion, and by way of summary, the literature review makes a compelling case that resilience is a theoretically ambiguous and multifaceted concept. Resilience is defined variously as:

- A process of adapting to systemic oppression (McLean et al., 2024).
- A resource-dependent outcome for stigmatized groups (King et al., 2024).
- A recovery of socioeconomic metrics (Sheetal et al., 2024).
- A state of positive outcomes across multiple domains (Bezek et al., 2024).
- A dynamic interplay of capacities and adaptation (Ahrens et al., 2024; Bergeman and Nelson, 2024).
- A process facilitated by social policies and community resources (Last et al., 2024; Walker et al., 2024).
- A protective mechanism rooted in familial identity and practices (Del Toro et al., 2024).

This diversity of definitions, contexts, and measurement tools confirms that “resilience” is less of a cohesive theoretical construct and more of a broad umbrella term used to describe a wide range of positive adaptations. The field is moving away from simplistic, individualistic views toward a more nuanced understanding that centers on context, resources, and systemic factors. However, until a new, unifying framework emerges, the term “resilience” will remain a theoretically fuzzy concept, its meaning contingent on the specific discipline and research question at hand.

The preceding literature review highlights a critical problem in the study of resilience: a profound lack of a unified theoretical framework. The diverse and often contradictory definitions—ranging from individual coping mechanisms and neurobiological markers to community-level resources and socioeconomic outcomes—are examples of definitional ambiguity that have given rise to significant conceptual and methodological limitations, calling into question the credibility and generalizability of existing research, as explicitly stated by Infurna et al. (2024).

Notably, a decade ago, Rabenu and Tziner (2016) shared Britt et al. (2016)concern over the confusion surrounding the conceptual definition of resilience, declaring that this thorny issue should be solved. Hence, building on the existing resilience literature, this commentary aims to clarify, to some extent, the ambiguous state of the resilience construct and, in parallel, to enhance the resilience construct's validity.

To that end, we recommend a definitional-formal framework of resilience, capitalizing on the facet analytical approach developed or introduced by Rabenu and Tziner (2016) and Tziner (1987). This approach posits that the components of a problem or an issue under investigation can be defined formally (Guttman, 1957). A facet is a criterion or a rule for classifying items associated with a given concept (Elizur, 1984; Roazzi et al., 2015). A natural way to define the structural configuration of a multi-component concept is to spell out the facets considered to exhaust its content (Elizur, 1984; Tziner, 1987). According to this approach, the content of a concept is broken down into facets that represent the most important properties of the concept domain (content). Facets are, therefore, a classification of elements of a concept's content, according to some rules (i.e., exclusive features).

This framework brings much-needed clarity by breaking down the concept “resilience” into four basic facets appearing in the literature—Facet A: *Modalities of coping*; Facet B: *Time span of resilient behavior*; Facet C: *Level of growth*; and Facet D: *Domain of outcome*. Specifically, these facets are defined as follows:

- Facet A—*Modalities of coping*—The modalities incorporate positive self-perceptions and a positive outlook on life as cognitive contributors to higher resilience as well as emotional stability and adaptation to adversity.
- Facet B—*Time span of the resilient behavior*—This facet describes the ability to maintain a stable equilibrium of normal functioning immediately after difficult events and, thereafter, for a while.
- Facet C—*Level of growth*—This facet addresses the ability to return to one's previous level of functioning or maintain a stable equilibrium of normal functioning after difficult events.
- Facet D *Domain of resilient outcome*—This facet measures the degree of positive outcomes following exposure to adversity.

Although the proposed approach has not yet been empirically tested, it has the potential to conceptualize resilience in a new and multidimensional way, something that has not been done before and deserves in-depth examination.

Furthermore, Rabenu and Tziner (2016) formally expressed the relationships between the facets in their mapping sentence definition of resilience (Figure 1).

**Figure 1:**
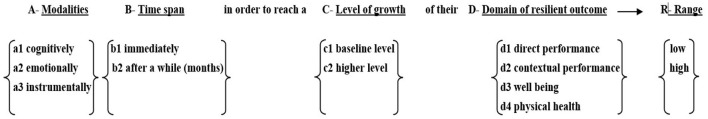
Mapping sentence definition of resilience.

This conceptualization comprises 3X2X2X4=48 components or elements. Assume that we conceive an instrument to assess resilience, incorporating at least two to three items (to ensure reasonable reliability) for each element. The assessment of resilience, drawing upon this definition, becomes quite cumbersome, and, in practice, questionable.

Perhaps, then, we may suffice sampling 48 components, a partial group representing the totality of facets adequately (a subset). Such a solution—applying the notion of sampling a population of individuals to sampling components in a conceptual space underlies the short versions of the instruments measuring the Big Five personality traits or the LMX leadership approach (Day and Miscenko, 2016).

This mapping sentence solution should be regarded as an open system. Empirical studies should be conducted (replications) to corroborate the veracity of the conceptualization of the construct of resilience, drawing upon the facet mapping sentence. If insufficient correspondence unfolds between the structure defined by the mapping sentence and the empirical reality, then the mapping sentence should be revised either by adding components or omitting some. The suggested structure embedded in the mapping sentence is not the ultimate truth, however. The structure, ultimately, should be regarded as a valuable solution to the thorny issue of conceptualizing resilience.

## Summary and conclusion

In this perspective article, we argue that the field of resilience is in a state of “definitional crisis,” with no unified theoretical framework. A meta-analysis by Good et al. (2025) demonstrates that while resilience is generally linked to positive workplace outcomes like performance and wellbeing, the inconsistent findings on the role of adversity make it hard to draw definitive conclusions.By reviewing previous literature, we discerned that traditional, individual-centric models of resilience have been widely criticized for failing to account for systemic factors like racism and capitalism. Several studies, including those by Walker et al. (2024); Last et al. (2024), propose multilevel frameworks that emphasize community resources and public policy as key determinants of resilience, shifting the focus from an internal trait to a social and economic outcome. Furthermore, other research defines resilience from various additional perspectives, such as a dynamic process (Ahrens et al., 2024) or even a purely economic metric (Sheetal et al., 2024). Acknowledging this confusion, we introduce Rabenu and Tziner (2016) proposed facet analytical approach as a (partial) solution to resolving the “definitional crisis,” thus creating a more formal and structured definition that enhances the construct validity and brings clarity to the field.

## Data Availability

The original contributions presented in the study are included in the article/supplementary material, further inquiries can be directed to the corresponding author.
